# Portuguese validation of the Cambridge pulmonary hypertension outcome review (CAMPHOR) questionnaire

**DOI:** 10.1186/s12955-016-0513-8

**Published:** 2016-07-26

**Authors:** Abílio Reis, James Twiss, Margarida Vicente, Fabienne Gonçalves, Luísa Carvalho, José Meireles, Alzira Melo, Stephen P. McKenna, Luís Almeida

**Affiliations:** 1 Pulmonary Vascular Disease Unit, Medicine Department, Centro Hospitalar do Porto-Hospital de Santo António, Largo Professor Abel Salazar, 4099-001 Porto, Portugal; 2 Galen Research Ltd, Manchester, UK; 3 Department of Health Sciences, University of Aveiro, Aveiro, Portugal; 4 Faculty of Medicine, Department of Pharmacology & Therapeutics, University of Porto, Porto, Portugal

**Keywords:** Pulmonary hypertension, Precapillary pulmonary hypertension, Pulmonary arterial hypertension, Quality of life, CAMPHOR, Portuguese adaptation, Nottingham Health Profile

## Abstract

**Background:**

Patients with pulmonary arterial hypertension (PAH) and other forms of precapillary pulmonary hypertension (PH) have impaired quality of life (QoL). The Cambridge Pulmonary Hypertension Outcome Review (CAMPHOR) is a PH-specific patient-reported outcome measure that assesses symptoms, activity limitations and QoL. It was originally developed in UK-English. The main objective of this study was to create an adaptation of the CAMPHOR suitable for a Portuguese-speaking population.

**Methods:**

A multi-step approach was followed: bilingual and lay panel translation; cognitive debriefing interviews; and psychometric testing in repeated postal surveys (2 weeks apart) including assessment of internal consistency, reproducibility and validity. The Nottingham Health Profile (NHP) questionnaire was used as a comparator instrument to test convergent validity.

**Results:**

The CAMPHOR was translated without difficulty by the two panels. Cognitive debriefing interviews showed the questionnaire was easily understood and considered relevant to patients’ experience with their illness. Psychometric evaluation was performed with 50 PAH patients (47 ± 14 years, 37 women). Cronbach’s alpha coefficients showed good internal consistency for the three CAMPHOR scales [Symptoms = 0.95; Activities = 0.93 and QoL = 0.94]. Test-retest coefficients showed that all scales had excellent reliability (Symptoms = 0.94; Activities = 0.89 and QoL = 0.93), indicating low levels of random measurement error. The CAMPHOR correlated as expected with the NHP. The magnitude of correlations followed a similar pattern to those in the original development study. The CAMPHOR also exhibited evidence of known group validity in its ability to distinguish between self-reported severity and general health groups.

**Conclusions:**

A valid and reliable version of the CAMPHOR questionnaire for the European Portuguese-speaking population was developed and is recommended for use.

**Electronic supplementary material:**

The online version of this article (doi:10.1186/s12955-016-0513-8) contains supplementary material, which is available to authorized users.

## Background

Precapillary pulmonary hypertension (PH) is characterized by an obstructive vasculopathy of the pulmonary circulation leading to increased pulmonary arterial pressure, pulmonary vascular resistance and, eventually, right-sided heart failure and death [1–4]. Multiple conditions and comorbidities are associated with PH [5]. Patients present unspecific symptoms, such as exertion dyspnoea, fatigue, chest pain, palpitations, oedema or syncope, resulting in frequently delayed diagnosis [4].

PH prognosis improved in recent years due to better understanding of PH pathobiology, availability of new drugs (endothelin receptor antagonists, phosphodiesterase-5 inhibitors, soluble guanylate cyclase stimulators and prostacyclin analogues) and renewed therapeutic strategies [6–9]. Still, a cure for pulmonary arterial hypertension (PAH) and other precapillary forms of PH is far from being available.

The impact of the disease and some types of treatment, like parenteral drugs or oxygen administration, can lead to impaired QoL, social isolation, as well as anxiety and depression [10]. Assessments of health-related quality of life (HRQoL) are currently recommended during global evaluation of PH patients, both in the context of clinical trials and routine clinical practice. Although some generic questionnaires such as the Short Form 36 Health Survey (SF-36) have shown prognostic value in PAH [11, 12], generally the use of generic questionnaires or those specific to other conditions has important limitations if they are not eventually validated in an appropriate PH sample [13]. Several attempts to evaluate other generic questionnaires, such as Nottingham Health Profile (NHP), and EuroQol Group 5-Dimension Self-Report Questionnaire (EQ-5D) or other disease-specific questionnaires, such as the Minnesota Living with Heart Failure Questionnaire (MLHFQ) or the Chronic Heart Failure questionnaire (CHQ), have failed to prove their specificity for the disease both in real life settings and clinical trials [14–21]. This lead to the development of disease-specific questionnaires such as the Cambridge Pulmonary Hypertension Outcome Review (CAMPHOR) [12, 13], and more recently, the emPHasis-10 [22] and the Pulmonary Arterial Hypertension-Symptoms and Impact (PAH-SYMPACT) [23], which are under evaluation. Disease-specific assessment instruments provide outcome measures that are more relevant to actual patient experience. Evidence also demonstrates that these outcomes are more sensitive to change than generic ones [24–29].

The CAMPHOR was originally developed and validated in the United Kingdom, [30] and subsequently adapted for use in the US, Canada, Australia/New Zealand, Sweden and Austria/Germany/Switzerland [13, 31–34]. It consists of three different scales: a 25-item Symptom scale, to assess energy, breathlessness and mood (low score indicates minimal symptoms); a 15-item Activity limitations scale with a 3-point rating system (score ranges from 0 to 30, lower score indicates minimal activity limitation); and a 25-item QoL scale (lower score indicates better QoL) [30].

This paper describes the Portuguese translation and validation of the CAMPHOR. The development of this language version will allow the use of CAMPHOR in routine clinical practice and in clinical trials in the European Portuguese-speaking population with PAH and other precapillary forms of PH.

## Methods

CAMPHOR translation and validation was based in three main stages: (1) bilingual and lay panel translation; (2) cognitive debriefing interviews; and (3) psychometric testing in repeated postal surveys, 2 weeks apart. Psychometric analyses included test-retest reliability, internal consistency, convergent validity, and known group validity. The study was approved by the Independent Ethics Committee of Centro Hospitalar do Porto and written informed consent was obtained from all participants before enrolment in the study.

- Step 1: Translation
  - The bilingual panel involved a group of six participants who had Portuguese as their primary language and were fluent in Portuguese and English. Participants were excluded if they had medical training, were a professional translator or if they were familiar with PH. The Principal Investigator lead the panel discussion and a representative of the original measure development team attended the meeting, advising in situations of difficult decision-making by explaining the original concept of the items. Each scale item was discussed until agreement was reached.
  - The main goal of this panel was to provide the initial translation of the questionnaire. When translating the content, emphasis was placed on achieving conceptual equivalence rather than providing a literal translation. In situations where agreement could not be reached, the panel provided alternative translations for later consideration by the lay panel.
  - A separate lay translation panel consisted of a group of five monolingual Portuguese-speaking participants with an average education level. The Portuguese translation of the questionnaire developed by the bilingual panel was presented and discussed. Participants were asked to comment on the language in terms of comprehension and acceptability. The main goal of this panel was to ensure that the language used in the questionnaire was adequate for respondents with average educational level.
- Step 2: Cognitive Debriefing Interviews
  - Face-to-face cognitive debriefing interviews were conducted with 10 patients in the PH outpatient clinic at Centro Hospitalar do Porto—Hospital Santo António (Table 1). The purpose of the interviews was to assess the relevance, acceptability, comprehensiveness and understandability of the questionnaire items.

**Table 1:** Demographic and baseline characteristics of the study samples

Parameter	Cognitive debriefing interview panel (*n* = 10)	Psychometric testing panel (*n* = 50)
Gender, *n* (%)		
Male	3 (30.0)	13 (26.0)
Female	7 (70.0)	37 (74.0)
Age, years		
Mean	47.8	46.8
Range	23–70	20–75
Marital Status, *n* (%)		
Single	3 (30.0)	12 (24.0)
Married/living as married	5 (50.0)	35 (70.0)
Divorced/Separated	2 (20.0)	3 (6.0)
Employment Status, *n* (%)		
Student	0 (0.0)	2 (4.0)
Full-time	2 (20.0)	16 (32.0)
Part-time	1 (10.0)	0 (0.0)
Unemployed	1 (10.0)	0 (0.0)
Retired	5 (50.0)	23 (46.0)
Long-term sick leave	1 (10.0)	0 (0.0)
Homemaker	0 (0.0)	9 (18.0)
PH aetiology, *n* (%)		
Idiopathic/Heritable PAH	3 (30.0)	12 (24.0)
Connective Tissue Disorders	1 (10.0)	7 (14.0)
Human Immunodeficiency Virus	0 (0.0)	1 (2.0)
Porto-Pulmonary Hypertension	0 (0.0)	3 (6.0)
Congenital Heart Disease	3 (30.0))	11 (22.0)
Interstitial Lung Disease	0 (0.0)	1 (2.0)
Chronic Thromboembolic Pulmonary Hypertension	3 (30.0)	12 (24.0)
PH with unclear multifactorial mechanisms	0 (0.0)	2 (4.0)
Mixed	0 (0.0)	1 (2.0)

*Abbreviations*: *PH* pulmonary hypertension
  - During the interviews, participants were asked to complete the questionnaire, comment on any aspect they thought had been omitted and answer some specific questions about the questionnaire.
- Step 3: Validation
  - To validate the Portuguese version of the CAMPHOR, 50 patients with precapillary PH were recruited in the outpatient clinic at Centro Hospitalar do Porto—Hospital Santo António, between 14SEP2012 and 16SEP2013. Their mean disease duration was 57.06 (SD = 58.81) months. During a programmed visit, participants were informed about the study methodology, signed an informed consent form and received packs containing written instructions, two samples of the CAMPHOR and NHP questionnaires (to be filled in at home, 2 weeks apart) and a pre-paid postal return envelope. All participants received unique identification numbers. Only returned completed surveys were considered for analysis.
  - Demographic (birth date, gender, marital and working status) and clinical data (PH aetiology, WHO/NYHA functional class, 6MWD, NT-proBNP, treatment) were gathered from the PH clinic dedicated informatics system (PAHTool^®^, Inovultus Lda, Portugal) and exported to Microsoft Excel.

### Statistical analysis

The distributional properties of the measures were explored through descriptive statistics [mean, standard deviation, median and interquartile range (IQR) and floor and ceiling effects (% of patients scoring the minimum and maximum scores)]. Scores were compared by age and gender.

Internal consistency was assessed using Cronbach’s alpha coefficients. Alpha measures the extent to which the items in a scale are inter-related. A low alpha (below 0.7) indicates that the items do not work together to form a scale [25]. In addition, item total correlations (ITCs) should be between 0.2 and 0.8.

The test-retest reliability of a measure is an estimate of its reproducibility over time when no change in the condition has taken place. It is calculated by correlating scores obtained on two different occasions. Spearman’s rank correlation coefficient was used for the analyses. For low random measurement error, a correlation coefficient of ≥0.85 is necessary (26).

Convergent validity is used to determine the level of association between scores on one scale and those on a comparator scale that measures the same or related constructs. For the present investigation, the NHP was used as a comparator instrument. Portuguese CAMPHOR scores were correlated with NHP scores by Spearman rank correlation coefficients.

Known group validity can be assessed by testing the ability of a measure to distinguish between groups of people that differ according to some known factor. The factors used for the present investigation were self-reported general health (poor, fair, good, very good) and severity of symptoms (mild, moderate and severe).

Non-parametric tests for independent samples (Mann-Whitney *U* Test for two groups or Kruskal-Wallis One-Way Analysis of Variance for three or more groups) were employed to test for differences in CAMPHOR scores between groups.

Statistical analyses were performed using IBM SPSS version 21.0.

## Results

### Translation

Equal numbers of men and women (3 males and 3 females) were included in the bilingual panel. The mean age of the participants was 37 years (range 21–52 years). Most of the translation process occurred with little discussion and disagreement between panel participants. The main challenge during the translation was the selection of words that would allow the items to be expressed colloquially.

The lay panel involved one male and four female participants with a mean age of 37 years (range 16–66 years). The panel reported that the language of the questionnaire was generally good, objective, direct, and clear. However, panel members suggested some alternative wording to improve understanding. For example, item 1 of the Symptoms scale was changed because the Lay Panel thought that “without strengths” rather than “low strengths” would be easier to understand.

### Cognitive debriefing interviews

All CAMPHOR questionnaires were completed within a mean of 17 min (standard deviation 10.5 min). In general, patients understood the questionnaire and assessed it as being simple and easy to complete. Three elderly, rural, low literacy patients needed supplementary information to fully understand the instructions. All patients found that the questionnaire reflected their health condition and daily activities. The questionnaire’s content was considered appropriate, relevant and comprehensive. No questionnaire items were identified as inappropriate or unacceptable.

### Validation

Fifty patients were recruited (Time 1) and 47 (94 %) completed and returned the questionnaires. Three subjects withdrew from the study, one was lost to follow up and two others were submitted to surgery between the surveys. The mean time between repeated post surveys was 14.2 days (median 14.0; *n* = 47). Main PH aetiologies are shown in Table 1.

### Sample demographics

Table 1 shows participants’ characteristics and Table 2 presents disease information at Time 1.

**Table 2:** Disease information of the Psychometric testing panel

Parameter	Psychometric testing panel (*n* = 50)
Self-reported general health, *n* (%)	
Poor	4 (8.3)
Fair	2 (4.2)
Good	11 (22.9)
Very Good	31 (64.6)
Self-reported severity of disease, *n* (%)	
Mild	6 (12.2)
Moderate	24 (49.0)
Severe	19 (38.8)
Flare up, *n* (%)	
No	41 (83.7)
Yes	8 (16.3)
Requirement of oxygen or aids, *n* (%)	
No	28 (57.1)
Yes	21 (42.9)

### Questionnaire descriptive scores

Descriptive statistics at Time 1 and Time 2 are shown in Table 3.

**Table 3:** Questionnaire descriptive statistics for Time 1 and Time 2

Parameter	*n*	Median (IQR)	Mean (SD)	Min–Max	% Max scoring	% Min scoring
Time 1						
*CAMPHOR*						
Symptoms	45	6.0 (3.0–15.5)	9.4 (7.9)	0.0–25.0	8.9	2.2
Activities	45	10.0 (6.0–16.0)	11.2 (6.4)	2.0–27.0	4.4	2.2
QoL	43	6.0 (1.0–14.0)	8.1 (7.3)	0.0–25.0	16.3	2.3
*NHP*						
Energy Scale	47	0.0 (0.0–33.3)	27.0 (37.2)	0.0–100.0	57.4	14.9
Pain Scale	48	0.0 (0.0–37.5)	20.3 (31.1)	0.0–100.0	58.3	2.1
Emotional Reactions	48	22.2 (11.1–44.4)	27.8 (24.8)	0.0–88.9	22.9	2.1
Sleep Scale	47	20.0 (0.0–60.0)	30.2 (34.6)	0.0–100.0	44.7	8.5
Social Isolation	49	0.0 (0.0–20.0)	13.9 (24.2)	0.0–80.0	71.4	2.0
Physical Mobility	47	12.5 (0.0–50.0)	26.3 (26.2)	0.0–87.5	29.8	2.1
NHP-D	44	3.0 (1.0–10.3)	5.4 (5.7)	0.0–20.0	18.2	2.3
Time 2						
*CAMPHOR*						
Symptoms	42	5.0 (1.8–16.3)	8.3 (7.9)	0.0–25.0	16.7	2.4
Activities	44	6.0 (4.0–12.0)	9.2 (7.2)	0.0–30.0	2.3	2.3
QoL	42	4.5 (0.8–13.0)	7.2 (7.1)	0.0–25.0	23.8	2.4
*NHP*						
Energy Scale	45	0.0 (0.0–33.3)	25.9 (37.5)	0.0–100.0	60.0	15.6
Pain Scale	42	0.0 (0.0–33.3)	18.5 (29.5)	0.0–100.0	57.1	2.4
Emotional Reactions	42	16.7 (0.0–33.3)	25.7 (28.8)	0.0–100.0	26.2	4.8
Sleep Scale	45	20.0 (0.0–60.0)	28.9 (34.8)	0.0–100.0	44.4	11.1
Social Isolation	43	0.0 (0.0–40.0)	15.3 (26.8)	0.0–100.0	69.8	2.3
Physical Mobility	44	25.0 (0.0–46.9)	26.4 (26.3)	0.0–100.0	29.5	2.3
NHP-D	39	3.0 (0.0–8.0)	5.3 (6.3)	0.0–23.0	25.6	2.6

*Abbreviations*: *CAMPHOR* Cambridge Pulmonary Hypertension Outcome Review, *IRQ* inter-quartile range, *Max* Maximum, *Min* minimum, *QoL* quality of life, *NHP* Nottingham Health Profile

Floor (>10 % of patients scoring minimum) effects were identified in the CAMPHOR QoL scale Time 1 and the CAMPHOR QoL and Symptoms scales at Time 2. However, this is likely to reflect the mild nature of a subgroup of the sample. A possible ceiling effect might have occurred, since 16.3 % of the patients reached the maximum score in the QoL scale at Time 1. These effects were observed in the NHP scales.

### Internal consistency and reproducibility

Cronbach’s alpha coefficients results are summarized in Table 4. The scales of Portuguese CAMPHOR showed excellent internal consistency with scales ranging between (0.93 and 0.95). Inter-item total correlations for each item are shown in Additional file 1: Tables S1, S2, S3. Reproducibility was above the required 0.85 level for all three CAMPHOR scales: Symptoms = 0.94, Activity limitations = 0.89 and QoL = 0.93.

**Table 4:** Cronbach’s Alpha coefficients for Time 1 and Time 2

Instruments	Reliability coefficient	
	Time 1 (*n* = 50)	Time 2 (*n* = 47)
*CAMPHOR*		
Symptoms	0.95	0.95
Activities	0.93	0.95
QoL	0.94	0.94

*Abbreviations*: *CAMPHOR* Cambridge Pulmonary Hypertension Outcome Review, *NHP* Nottingham Health Profile, *QoL* quality of Life

### Convergent reliability

Table 5 shows the correlation between CAMPHOR and NHP (six sections and NHP-D) scales at Time 1. High correlations were observed between CAMPHOR symptoms and Emotional reactions, Physical mobility and Energy showing the importance of these factors on PH symptomatology. As expected, CAMPHOR Activities correlated more strongly with Physical mobility. The QoL scale correlated more strongly with Energy, Emotional reactions, Physical mobility and Overall distress, indicating that multiple factors influence QoL in PH.

**Table 5:** Correlation coefficients between CAMPHOR scales and NHP

Parameters	Symptoms (*n* = 50)	Activities (*n* = 50)	QoL (*n* = 50)
*NHP*			
Energy Scale	0.82^a^	0.76^a^	0.75^a^
Pain Scale	0.66^a^	0.67^a^	0.56^a^
Emotional Reactions	0.78^a^	0.66^a^	0.78^a^
Sleep Scale	0.40^a^	0.47^a^	0.55^a^
Social Isolation	0.52^a^	0.40^a^	0.61^a^
Physical Mobility	0.83^a^	0.84^a^	0.77^a^
NHP-D	0.80^a^	0.72^a^	0.82^a^

*Abbreviations*: *CAMPHOR* Cambridge Pulmonary Hypertension Outcome Review, *NHP* Nottingham Health Profile, *QoL* quality of life

^a^Correlation is significant at the 0.01 level

### Association of CAMPHOR scores with demographic factors

No significant differences in CAMPHOR scores were found between participants grouped by age (above versus below median age) or gender.

### Known group validity

Table 6 shows the results of the known groups analyses. Significant differences in ASQoL scores were observed between patients grouped by overall general health and perceived severity of disease.

**Table 6:** Mean scores by known groups

Known groups	Symptoms		Activities		QoL	
	*n*	Mean (SD)	*n*	Mean (SD)	*n*	Mean (SD)
*General Health*						
Very Good/Good	13	4.5 (4.6)	11	6.3 (2.9)	13	3.8 (5.3)
Fair/Poor	31	11.4 (8.2)	33	12.7 (6.6)	29	10.0 (7.5)
*P-value*	–	<0.01	–	<0.01	–	<0.05
*Self-reported severity of disease*						
Mild/Moderate	27	7.3 (7.1)	27	9.3 (5.6)	26	5.9 (6.3)
Severe/Very Severe	18	12.6 (8.2)	18	14.0 (6.7)	17	11.5 (7.5)
*P-value*	–	<0.05	–	<0.01	–	<0.05

*Abbreviations*: *QoL* quality of life, *SD* standard deviation

## Discussion

Current guidelines recommend initial and follow-up disease severity and QoL assessments to support decisions regarding PH treatment [35]. Previous validation of the CAMPHOR questionnaire in other geographical and cultural contexts demonstrated its superior specificity for PH versus general questionnaires, such as NHP, SF-36 or LHFQ. Furthermore, the development of the CAMPHOR utility index added the possibility of cost-utility analyses, which is particularly relevant for PH disease management [36].

Our study demonstrates that our European Portuguese version of CAMPHOR is a valid, internally consistent and reliable patient-reported outcome measure for the European Portuguese-speaking population with precapillary PH. This study was conducted exclusively in Portugal, therefore the use of our version in other Portuguese-speaking countries cannot be recommended, due to significant differences in culture, literacy and language (both in terms of semantics and vocabulary).

Most of the patients in the cognitive debriefing panels reported that the instrument was easy to complete and all patients reported that the questionnaire covered all important aspects of their experience with the disease. Three patients needed, nonetheless, additional information to fully understand the instructions during the cognitive debriefing interviews; these were older patients, from rural settings and with low literacy skills, and after additional clarifications, they finally were able to understand the instructions. In our study, patients took a mean of 17 min to complete the questionnaire, which is substantially longer than patients in the original instrument (10 min) [30] and more in line with data from the German translation (15 min) [34]. We hypothesise that this longer completion time could be associated with lower literacy and higher proportions of patients from rural contexts in our population, however, literacy was not evaluated in our sample hampering further clarification.

In this study, the proportion of patients reaching maximum scores in the CAMPHOR QoL scale was surprisingly high, particularly when compared to previous validation studies (16.3 % vs. 0.0–0.7 % in other studies [13, 30–34]), which could be suggestive of a substantial ceiling effect. These results, could potentially be explained by disease severity, comorbidities, oxygen use, or even literacy. However, the proportion of patients reaching maximum scores is actually similar (or even higher) in the NHP comparator scale, which could be indicative of a tendency to the extremes in this population rather than a substantial ceiling effect. Given the similar results observed in the NHP comparator scale we do not envision this possible ceiling effect as a major limitation to the clinical use of this European Portuguese translation of CAMPHOR.

We found significant relationships between the CAMPHOR scales and the relevant domains of the NHP and the magnitude of the correlations followed a similar pattern to those described in the original development study [30]. The CAMPHOR also showed evidence of known group validity in its ability to distinguish between groups of patients known to differ between self-ratings of disease severity and general health. The study showed that patients with precapillary PH experienced diminished QoL and increased symptoms and functional limitations. Results are consistent with the findings that patients with PH experience impairment in HRQoL [32, 33].

The major limitation of the present study is the small sample size, but given the low prevalence of precapillary PH related disease it can be considered an adequate sample for the purpose. Further studies to demonstrate CAMPHOR clinical validity, namely correlations with NYHA/WHO functional class, 6MWD and biomarkers, in the Portuguese population are being conducted.

## Conclusions

In conclusion, this study gave sufficient evidence that our adaptation of CAMPHOR is an effective and consistent tool in European Portuguese-speaking population with precapillary PH. Incorporating this questionnaire in future clinical trials and especially in clinical practice will improve our global clinical evaluation of the PH patient and will improve knowledge of the health impact of PH. Routine HRQoL evaluation at first presentation and regularly during follow-up can help to educate/familiarize the patient with these tools and help physicians make evidence-based decisions.

## Abbreviations

6MWD, 6-min waling distance; CAMPHOR, Cambridge Pulmonary Hypertension Outcome Review; HRQoL, health-related quality of life; IQR, interquartile range; ITC, item total correlation; MLHFQ, Minnesota Living with Heart Failure Questionnaire; NHP, Nottingham Health Profile; NYHA/WHO, New York Heart Association/World Health Organisation; PAH, pulmonary arterial hypertension; PH, pulmonary hypertension; QoL, quality of life; SF-36, short form 36 health survey

## Additional file

Additional file 1: Supplementary Table 1. Item reliability statistics for Symptoms scale. Supplementary Table 2. Item reliability statistics for Activities scale. Supplementary Table 3. Item reliability statistics for Quality of life (QoL) scale. (DOC 294 kb)
