# Supplementary material for: Portuguese validation of the Cambridge pulmonary hypertension outcome review (CAMPHOR) questionnaire
Source: Health Qual Life Outcomes. 2016 Jul 26;14:110. doi: 10.1186/s12955-016-0513-8 (PMC4962538; doi:10.1186/s12955-016-0513-8)
Supplement: Additional file 1: — Supplementary Table 1. Item reliability statistics for Symptoms scale. Supplementary Table 2. Item reliability statistics for Activities scale. Supplementary Table 3. Item reliability statistics for Quality of life (QoL) scale. (DOC 294 kb) [file 12955_2016_513_MOESM1_ESM.doc]

**Additional File 1**

**Supplementary Table 1.** Item reliability statistics for Symptoms scale.

| **Item** |  | **Time 1** |  |  | **Time 2** |  |
| --- | --- | --- | --- | --- | --- | --- |
|  | **Scale Mean if Item Deleted** | **Corrected Item-Total Correlation** | **Alpha if Item Deleted** | **Scale Mean if Item Deleted** | **Corrected Item-Total Correlation** | **Alpha if Item Deleted** |
| Item 1 | 8.98 | 0.666 | 0.947 | 7.95 | 0.720 | 0.951 |
| Item 2 | 8.83 | 0.544 | 0.949 | 7.77 | 0.620 | 0.952 |
| Item 3 | 9.17 | 0.688 | 0.947 | 8.00 | 0.685 | 0.952 |
| Item 4 | 8.80 | 0.656 | 0.948 | 7.81 | 0.795 | 0.950 |
| Item 5 | 9.20 | 0.772 | 0.946 | 8.09 | 0.774 | 0.951 |
| Item 6 | 9.24 | 0.669 | 0.948 | 8.05 | 0.755 | 0.951 |
| Item 7 | 9.28 | 0.582 | 0.948 | 8.23 | 0.497 | 0.954 |
| Item 8 | 9.17 | 0.646 | 0.948 | 8.05 | 0.568 | 0.953 |
| Item 9 | 9.00 | 0.760 | 0.946 | 7.95 | 0.748 | 0.951 |
| Item 10 | 9.09 | 0.762 | 0.946 | 8.00 | 0.800 | 0.950 |
| Item 11 | 9.22 | 0.673 | 0.947 | 8.19 | 0.579 | 0.953 |
| Item 12 | 9.15 | 0.672 | 0.947 | 8.09 | 0.614 | 0.952 |
| Item 13 | 8.87 | 0.700 | 0.947 | 7.74 | 0.721 | 0.951 |
| Item 14 | 9.04 | 0.748 | 0.946 | 8.00 | 0.678 | 0.952 |
| Item 15 | 9.11 | 0.628 | 0.948 | 8.02 | 0.677 | 0.952 |
| Item 16 | 8.80 | 0.568 | 0.949 | 7.72 | 0.664 | 0.952 |
| Item 17 | 9.30 | 0.657 | 0.948 | 8.19 | 0.629 | 0.952 |
| Item 18 | 8.80 | 0.606 | 0.948 | 7.72 | 0.624 | 0.952 |
| Item 19 | 8.87 | 0.466 | 0.950 | 7.88 | 0.345 | 0.956 |
| Item 20 | 9.11 | 0.792 | 0.946 | 8.02 | 0.810 | 0.950 |
| Item 21 | 9.15 | 0.638 | 0.948 | 8.02 | 0.780 | 0.951 |
| Item 22 | 9.02 | 0.637 | 0.948 | 7.91 | 0.708 | 0.951 |
| Item 23 | 9.26 | 0.685 | 0.947 | 8.14 | 0.650 | 0.952 |
| Item 24 | 9.00 | 0.592 | 0.948 | 7.84 | 0.567 | 0.953 |
| Item 25 | 8.96 | 0.311 | 0.952 | 7.86 | 0.526 | 0.954 |

**Supplementary Table 2.** Item reliability statistics for Activities scale.

| **Item** |  | **Time 1** |  |  | **Time 2** |  |
| --- | --- | --- | --- | --- | --- | --- |
|  | **Scale Mean if Item Deleted** | **Corrected Item-Total Correlation** | **Alpha if Item Deleted** | **Scale Mean if Item Deleted** | **Corrected Item-Total Correlation** | **Alpha if Item Deleted** |
| Item 1 | 10.50 | 0.663 | 0.930 | 8.77 | 0.727 | 0.951 |
| Item 2 | 10.89 | 0.694 | 0.928 | 8.95 | 0.830 | 0.949 |
| Item 3 | 10.91 | 0.700 | 0.928 | 8.98 | 0.729 | 0.951 |
| Item 4 | 10.00 | 0.626 | 0.931 | 9.05 | 0.521 | 0.955 |
| Item 5 | 9.93 | 0.697 | 0.929 | 8.89 | 0.854 | 0.948 |
| Item 6 | 10.57 | 0.743 | 0.927 | 8.68 | 0.878 | 0.947 |
| Item 7 | 10.35 | 0.779 | 0.925 | 8.43 | 0.786 | 0.949 |
| Item 8 | 10.46 | 0.711 | 0.927 | 8.48 | 0.762 | 0.950 |
| Item 9 | 10.70 | 0.712 | 0.928 | 8.86 | 0.785 | 0.950 |
| Item 10 | 10.89 | 0.524 | 0.932 | 9.09 | 0.776 | 0.951 |
| Item 11 | 10.48 | 0.745 | 0.927 | 8.59 | 0.814 | 0.949 |
| Item 12 | 9.91 | 0.608 | 0.930 | 7.98 | 0.586 | 0.954 |
| Item 13 | 9.80 | 0.642 | 0.930 | 7.84 | 0.661 | 0.952 |
| Item 14 | 10.61 | 0.702 | 0.928 | 8.70 | 0.833 | 0.948 |
| Item 15 | 9.83 | 0.665 | 0.929 | 7.89 | 0.662 | 0.952 |

**Supplementary Table 3.** Item reliability statistics for Quality of life (QoL) scale.

| **Item** |  | **Time 1** |  |  | **Time 2** |  |
| --- | --- | --- | --- | --- | --- | --- |
|  | **Scale Mean if Item Deleted** | **Corrected Item-Total Correlation** | **Alpha if Item Deleted** | **Scale Mean if Item Deleted** | **Corrected Item-Total Correlation** | **Alpha if Item Deleted** |
| Item 1 | 7.93 | 0.672 | 0.936 | 7.23 | 0.376 | 0.940 |
| Item 2 | 7.89 | 0.434 | 0.939 | 7.07 | 0.508 | 0.939 |
| Item 3 | 7.91 | 0.486 | 0.938 | 7.18 | 0.387 | 0.940 |
| Item 4 | 8.05 | 0.578 | 0.938 | 7.14 | 0.554 | 0.938 |
| Item 5 | 7.89 | 0.450 | 0.939 | 7.05 | 0.583 | 0.938 |
| Item 6 | 8.00 | 0.634 | 0.937 | 7.02 | 0.648 | 0.937 |
| Item 7 | 7.89 | 0.536 | 0.938 | 7.11 | 0.674 | 0.937 |
| Item 8 | 8.00 | 0.555 | 0.938 | 7.09 | 0.467 | 0.939 |
| Item 9 | 7.82 | 0.493 | 0.938 | 6.91 | 0.589 | 0.938 |
| Item 10 | 7.64 | 0.664 | 0.936 | 6.91 | 0.786 | 0.935 |
| Item 11 | 7.77 | 0.651 | 0.936 | 7.02 | 0.673 | 0.937 |
| Item 12 | 7.75 | 0.691 | 0.936 | 6.98 | 0.675 | 0.936 |
| Item 13 | 7.68 | 0.712 | 0.935 | 7.00 | 0.584 | 0.938 |
| Item 14 | 7.86 | 0.687 | 0.936 | 7.00 | 0.584 | 0.938 |
| Item 15 | 7.70 | 0.746 | 0.935 | 6.84 | 0.701 | 0.936 |
| Item 16 | 7.80 | 0.517 | 0.938 | 6.98 | 0.683 | 0.936 |
| Item 17 | 7.57 | 0.640 | 0.936 | 6.75 | 0.606 | 0.938 |
| Item 18 | 7.70 | 0.703 | 0.935 | 6.89 | 0.772 | 0.935 |
| Item 19 | 7.82 | 0.649 | 0.936 | 6.98 | 0.588 | 0.938 |
| Item 20 | 7.50 | 0.653 | 0.936 | 6.64 | 0.662 | 0.937 |
| Item 21 | 7.57 | 0.626 | 0.937 | 6.73 | 0.642 | 0.937 |
| Item 22 | 7.98 | 0.550 | 0.938 | 7.18 | 0.554 | 0.938 |
| Item 23 | 7.91 | 0.575 | 0.937 | 7.00 | 0.513 | 0.939 |
| Item 24 | 7.89 | 0.552 | 0.938 | 6.98 | 0.636 | 0.937 |
| Item 25 | 7.77 | 0.564 | 0.937 | 6.89 | 0.542 | 0.938 |
